# Association of the *DNMT3B* -579G>T Polymorphism with Risk of Thymomas in Patients with Myasthenia Gravis

**DOI:** 10.1371/journal.pone.0080846

**Published:** 2013-11-19

**Authors:** Fabio Coppedè, Roberta Ricciardi, Maria Denaro, Anna De Rosa, Carlo Provenzano, Emanuela Bartoccioni, Angelo Baggiani, Marco Lucchi, Alfredo Mussi, Lucia Migliore

**Affiliations:** 1 Department of Translational Research and New Technologies in Medicine and Surgery, University of Pisa, Pisa, Italy; 2 Division of Neurology, Department of Neuroscience, University of Pisa, Pisa, Italy; 3 Division of Thoracic Surgery, Cardiac and Thoracic Department, University of Pisa, Pisa, Italy; 4 Institute of General Pathology, Catholic University of Rome, Rome, Italy; Università di Napoli Federico II, Italy

## Abstract

Increasing evidence suggests a contribution of epigenetic processes in promoting cancer and autoimmunity. Myasthenia gravis (MG) is an autoimmune disease mediated, in approximately 80% of the patients, by antibodies against the nicotinic acetylcholine receptor (AChR+). Moreover, epithelial tumours (thymomas) are present in about 10-20% of the patients, and there is indication that changes in DNA methylation might contribute to the risk and progression of thymomas. However, the role of epigenetics in MG is still not completely clarified. In the present study we investigated if a common polymorphism (-579G>T: rs1569686) in the promoter of the *DNMT3B* gene coding for the DNA methyltransferase 3B, an enzyme that mediates DNA methylation, increases the risk to develop MG or MG-associated thymomas. The study polymorphism was selected based on recent reports and a literature meta-analysis suggesting association with increased risk of various types of cancer. We screened 324 AChR+ MG patients (140 males and 184 females, mean age 56.0 ± 16.5 years) and 735 healthy matched controls (294 males and 441 females, mean age 57.3 ± 15.6 years). 94 of the total MG patients had a thymoma. While there was no association with the whole cohort of MG patients, we found a statistically significant association of the *DNMT3B* -579T allele (OR = 1.51; 95% CI=1.1-2.1, *P* = 0.01) and the TT homozygous genotype (OR = 2.59; 95% CI=1.4-4.9, *P* = 0.006) with the risk of thymoma. No association was observed in MG patients without thymoma, even after stratification into clinical subtypes. Present results suggest that the *DNMT3B* -579T allele might contribute to the risk of developing thymoma in MG patients, particularly in homozygous TT subjects.

## Introduction

Increasing evidence suggests a contribution of epigenetic modifications in complex diseases such as cancer and autoimmune disorders [1-3]. The term epigenetics refers to heritable and reversible modifications that alter gene expression without resulting in direct changes of the primary DNA sequence. Several epigenetic mechanisms are known, including DNA methylation, covalent modifications of histone tails, and nucleosome positioning, all interacting to determine chromatin folding and the relative accessibility of a given genetic locus to activating and suppressing transcription factors [4], and non coding RNAs affecting gene expression levels [5].

DNA methylation represents one of the most studied epigenetic marks for gene regulation; it consists of the addition of a methyl group to the 5’ position of the cytosine pyrimidine ring (5-methylcytosine) mediated by DNA methyltransferase enzymes (DNMTs) using *S*-adenosylmethionine (SAM) as the methyl donor compound [6]. Methylation of CpG rich regions (CpG islands) in the promoter of a given gene is commonly associated with gene silencing [6]. Most of the studies performed so far in autoimmune diseases have focused on DNA methylation impairments in systemic lupus erythematosus and rheumatoid arthritis [3]. There is however increasing evidence of methylation changes and/or impaired DNMTs activity in other autoimmune disorders such as Sjögren’s syndrome, multiple sclerosis, systemic sclerosis, psoriasis, and autoimmune thyroid diseases [3,7,8].

Myasthenia Gravis (MG) is a prototypic antibody-mediated autoimmune disease. In approximately 80% of the patients the disease is mediated by auto-antibodies against the nicotinic acetylcholine receptor (AChR) [9]. Despite emerging evidence of epigenetic changes in promoting autoimmunity, the role of epigenetics in MG is still scarcely investigated [10]. Moreover, MG is often associated with pathological changes of the thymus: thymic epithelial tumours are present in about 10-20% of MG patients, while up to 80% of the patients with early onset of disease have thymic hyperplasia [11]. Thymoma is an uncommon neoplasm derived from thymus epithelial cells, and studies aimed at analyzing DNA methylation of tumour-related genes showed altered methylation patterns of many of them, including among others *MGMT*, *hMLH1*, *p16/INK4*, and *RASSF1A*, whose increased methylation significantly correlated with tumour severity [12,13].

The goal of the present study was to investigate if a common polymorphism (-579G>T: rs1569686) in the promoter of the *DNMT3B* gene coding for the DNA methyltransferase 3B, one of the key *de novo* enzymes mediating DNA methylation, increases the risk to develop MG or MG-associated thymomas. The selected polymorphism has been suggested to impair DNA methylation of tumor suppressor genes, therefore impairing their expression levels, and for this reason it was investigated as a susceptibility factor for several cancer types [16-21]. A meta-analysis of published studies revealed that the mutant allele (T) is associated with increased risk of cancer compared to the wild type one (G) [22]. Moreover, *DNMT3B* promoter polymorphisms have been associated with global DNA methylation and are increasingly investigated in other complex diseases than cancer [23-27]. Indeed, clinical studies showed association with wide-spread DNA methylation and suicide attempts in psychiatric patients [23], with increased risk of early-onset schizophrenia [24], with risk of giving birth to a child with Down syndrome [25], as well as with increased risk of the autoimmune disease oral lichen planus [26], and with the progression of joint destruction in rheumatoid arthritis [27]. 

## Materials and Methods

### Study population

A total of 324 AChR+ MG patients and 735 healthy matched unrelated controls were recruited at the Myasthenia Clinic (Department of Neuroscience and Cardiac and Thoracic Department) of the Pisa University Hospital, at the Institute of General Pathology of the Catholic University of Rome, and at the Department of Translational Research and New Technologies in Medicine and Surgery of the University of Pisa. Disease diagnosis was based on characteristic signs and symptoms of MG coupled with an anti-AChR antibody positive test; patients with an anti-AChR antibody negative test were excluded from the present study. The main clinical characteristics of MG patients are listed in Table 1. The mean age (±S.D.) of the patients was 56.0 (± 16.5) years. According to disease onset age, patients were divided into early onset (≤45 years) and late onset (>45 years). According to the Osserman classification, they could be divided into pure ocular (class I) and generalized MG (class IIA, IIB, III, IV). MG was associated with different autoimmune disorders (AID) in 51 (15.7 %) out of 324 patients (see Table 1 for details). All patients had computed tomography (CT) scans of the chest and thymectomy was performed in 179 out of 324 patients according to the CT scan findings. Overall 94 patients (29.0%) had a thymoma. Thymic hyperplasia was found in 95 cases (29.3%), and a normal (involuted) thymus in 135 cases (41.7%). The control group includes 735 healthy volunteer subjects with no history of cancer or autoimmune diseases matched for age and gender with the patients (Table 1). All individuals were white Caucasians of Italian descent as determined by grandparents origin; each subject gave an informed written consent for genotype analysis before blood drawing. The study was performed in accordance to the Declaration of Helsinki, and was approved by the Ethics Committees of the Pisa University Hospital and of the Catholic University of Rome.

**Table 1:** Demographic characteristics of Myasthenia Gravis patients and Controls.

**Characteristics**	**MG patients, Total No. = 324**	**Controls, Total No. = 735**
Gender**^*a*^**: Females (%) / Males (%)	184 (56.8) / 140 (43.2)	441 (60.0) / 294 (40.0)
Age**^*b*^** (years): Mean ± S.D.	56.0 ± 16.5	57.3 ± 15.6
Age at onset (years): ≤ 45 (%) / > 45 (%)	170 (52.5) / 154 (47.5)	
Antibodies anti-AchR+	324 (100%)	
Osserman classification: Grade I	56 (17.3%)	
Osserman classification: Grade IIA	78 (24.1%)	
Osserman classification: Grade IIB	182 (56.2%)	
Osserman classification: Grade III	3 (0.9%)	
Osserman classification: Grade IV	5 (1.5%)	
Associated autoimmune diseases (AID)**^*c*^**	51 (15.8%)	
Thymic histology: normal thymus	135 (41.7%)	
Thymic histology: thymoma	94 (29.0 %)	
Thymic histology: hyperplasia	95 (29.3 %)	

*a* Gender: no significant difference between MG patients and controls

*b* Age: no significant difference between MG patients and controls

*c* Associated autoimmune diseases (AID): Hashimoto’s thyroiditis: 16 cases; Graves-Basedow disease: 14 cases; Type 1 Diabetes: 7 cases; Others: 14 cases.

### Genotyping procedures

Genomic DNA was extracted from peripheral blood lymphocytes by means of the QIAamp® Blood Mini Kit (Quiagen, Milan, Italy) according to the manufacturer’s protocol. Genotyping analysis was performed by means of PCR-RFLP techniques following the protocol of Lee et al. [28] with minor modifications [29]. Briefly, a 225 bp product was amplified using 1.25 Units of Taq DNA polymerase (Invitrogen, Milan, Italy), 10 pmol of each primer (forward: 5’-GAG GTC TCA TTA TGC CTA GG-3’ and reverse: 5’-GGG AGC TCA CCT TCT AGA AA-3’), 0.15mM of each dNTPs, 1.5mM MgCl2, and 40 ng of genomic DNA in a total volume of 25µl. PCR conditions were 35 cycles of 30s at 95°C, 45s at 56°C, and 45s at 72°C, preceded by an initial denaturation step of 5 min at 95°C and followed by a final elongation step of 10 min at 72°C. Overnight digestion at 37°C with *Pvu II* (Fermentas, Milan, Italy) resulted in an undigested 225-bp product for the wild type allele (G), and in 132- and 93-bp products for the mutant allele (T). Digestion products were visualized after electrophoresis on a 2% agarose gel containing ethidium bromide. Samples with previously confirmed wild-type, heterozygous and mutant genotypes [29] have been used as internal quality controls during PCR-RFLP reactions.

### Statistical analyses

Differences in mean age and gender between groups were evaluated by means of the Student’s T-test, and Fisher’s exact test, respectively. To verify that genotype frequencies were in Hardy-Weinberg equilibrium in controls we used Chi-square (χ^2^) analysis. Differences in allele and genotype distributions between groups have been evaluated by means of Fisher’s exact test. Odds ratios (ORs) have been calculated by means of unconditional logistic regression and given with 95% confidence intervals (CIs). All individual values were analyzed with the SPSS 16.0 statistical package for Windows. The statistical power of the study was evaluated by means of the statistical package QUANTO 1.2.4.exe. Given a case-control cohort of at least 300 cases and 700 controls, and a minor allele frequency (MAF) of 31% in Controls, the study had an a priory power of 82% to detect differences in allele frequencies.

## Results

Allele and genotype frequencies generated by the *DNMT3B* -579G>T polymorphism in patients and controls are shown in Tables 2 and 3, respectively . Genotype distributions in controls conformed to Hardy-Weinberg equilibrium (*P* = 0.18). No difference in allele and genotype frequencies was observed between patients and controls when MG patients were considered as a whole (Tables 2 and 3). After stratification of MG patients into three subgroups according to thymic pathology (normal thymus, thymic hyperplasia and thymoma), the comparison of each of the three subgroups with controls revealed a significant association of the *DNMT3B* -579T allele with increased thymoma risk (OR = 1.5; 95% CI = 1.1-2.1, *P* = 0.01). Moreover, the homozygous TT genotype was associated with an increased thymoma risk compared to the homozygous GG one (OR = 2.59; 95% CI = 1.4-4.9, *P* = 0.0059). Both associations remained significant even after Bonferroni’s correction for multiple comparisons (Tables 2 and 3). Given the number of thymoma cases (94) and controls (735), a post-hoc power analysis revealed that the study had >90% power to detect differences in allele frequencies. No difference in allele or genotype distributions was observed between MG patients with thymic hyperplasia and controls, or between MG patients with normal thymus and controls. 

**Table 2:** DNMT3B -579G>T allele frequencies in Myasthenia Gravis patients and Controls.

**Alleles**	**Controls, No. (%)**	**MG (Total), No. (%)**	**MG (Thymomas), No. (%)**	**MG (Hyperplasia), No. (%)**	**MG (Normal Thymus), No. (%)**
Allele G	1007 (68.5)	426 (65.7)	111 (59.0)	127 (66.8)	188 (69.6)
Allele T	463 (31.5)	222 (34.3)	77 (41.0)	63 (33.2)	82 (30.4)
OR (95%CI) T vs G	--	1.13 (.93-1.4) **^*a*^**	**1.51 (1.1-2.1) ^*a*^**	1.08 (.78-1.5) **^*a*^**	0.95(.72-1.3) **^*a*^**
*P*-value	--	0.226	**0.010^*b*^**	0.679	0.775

^***a***^ Compared to the control group

^***b***^ Fisher’s exact test *P*-value = 0.01; Bonferroni’s corrected *P*-value = 0.04

**Table 3:** DNMT3B -579G>T genotype frequencies in Myasthenia Gravis patients and Controls.

**Genotypes**	**Controls, No. (%)**	**MG (Total), No. (%)**	**MG (Thymomas), No. (%)**	**MG (Hyperplasia), No. (%)**	**MG (Normal Thymus), No. (%)**
GG	337 (45.8)	148 (45.7)	34 (36.2)	46 (48.4)	68 (50.4)
GT	333 (45.3)	130 (40.1)	43 (45.7)	35 (36.8)	52 (38.5)
TT	65 (8.9)	46 (14.2)	17 (18.1)	14 (14.8)	15 (11.1)
OR: GT vs. GG	--	0.89 (.67-1.2) **^*a*^**	1.28 (.80-2.1) **^*a*^**	0.77 (.48-1.2) **^*a*^**	0.77 (.52-1.1) **^*a*^**
OR : TT vs. GG	--	1.61 (1.1-2.5) **^*a*^**	**2.59 (1.4-4.9)*^a^*,*^b^***	1.58 (.82-3.0) **^*a*^**	1.14 (.62-2.1) **^*a*^**

^***a***^ OR and (95%CI) compared to the control group

^***b***^ Fisher exact Test *P*-value = 0.0059; Bonferroni’s corrected *P*-value = 0.04

We also compared allele and genotype frequencies generated by the *DNMT3B* -579G>T polymorphism in MG patients stratified according to disease age at onset (≤45 years vs. > 45 years), to disease severity (less severe forms: Grade I + IIA vs. more severe forms: Grade IIB + III + IV), or to the presence/absence of associated autoimmune diseases (associated AID vs. no associated AID). No difference in allele and genotype frequencies was observed in young onset vs. late onset MG patients, in less severe vs. more severe MG, or in patients with associated AID with respect to those with no associated AID (data not shown). 

## Discussion

In order to shed some light on the role of epigenetics in both MG and MG-associated diseases, we analyzed the *DNMT3B* -579G>T polymorphism in a large population of MG patients of Italian descent. Overall, no association of the studied polymorphism with MG was observed, even after stratification of the patients according to different clinical parameters like age at onset, disease severity according to Osserman classification, or presence/absence of other autoimmune diseases. However, when we stratified MG patients according to thymus pathology, we found a statistically significant association of the T allele with the presence of thymoma, particularly among carriers of the homozygous TT genotype. 

Present data are in agreement with a literature meta-analysis suggesting an increased risk of cancer associated with the presence of the *DNMT3B* -579T allele [22]. That meta-analysis included ten studies performed in Asian populations, and one single study performed in Caucasians. After stratification into ethnic groups, the authors observed association with cancer risk in Asians, likely because almost all the available studies had been performed in that ethnic group [22]. Interestingly, the frequency of the *DNMT3B* -579G allele in Asians is much lower than that of the *DNMT3B* -579T one [22]. However, to the best of our knowledge, previous studies have assessed the role of this polymorphism in many different cancers, including those of the gastro-intestinal and respiratory apparatuses, head and neck carcinomas, and nasopharyngeal carcinomas [14-21], but never in thymoma, either alone or associated to MG. 

Hirose and coworkers [12] investigated DNA methylation in 26 thymomas and 6 thymic carcinomas to clarify the association between aberrant DNA methylation and the clinico-pathological features of thymic neoplasms. They observed that almost 50% of thymic epithelial tumours showed aberrant methylation, and that the frequency of tumour methylation was correlated with their malignant behaviour. Moreover, also the number of hypermethylated genes increased with increasing malignant behaviour, suggesting that promoter hypermethylation, and the consequent down-regulation of specific tumour suppressor genes, may play an important role in the development of aggressive and invasive thymic epithelial tumours [12]. Their results were in accordance with Chen and coworkers, who found that tumor suppressor genes and DNA repair genes, including *hMLH1, RASSF1A*, *MGMT*, *p16/INK4*, *DAPK*, *FHIT*, RAR2, *CDH1*, and *APC*, are frequently hypermethylated in thymic epithelial tumours, all found to be methylated in almost 20 to 40% of the cases [13,30]. Several studies reported that DNMTs are upregulated in thoracic neoplasms, and that those cancers are characterized by global DNA hypomethylation and gene specific hypermethylation (reviewed in 30), and an inverse correlation between DNMT3B levels and global DNA methylation was observed in thymic epithelial tumours [13]. 

The functional role of the *DNMT3B* -579G>T polymorphism is not yet completely elucidated. Some authors suggest that it can directly impair promoter activity and gene expression levels, and others observed a linkage disequilibrium between rs1569686 and other *DNMT3B* promoter polymorphisms, namely −149C>T (rs2424913) and -283T>C (rs6058870), which have been functionally associated with promoter activity and gene expression levels [22,25,28,31]. Taken overall, results from those studies coupled with the evidence of an association of the *DNMT3B* -579G>T polymorphism with various types of cancer, suggest that this polymorphism might either have a functional role on gene expression levels, or be a tag SNP of functional haplotypes [22,28,31]. In this regard, several investigators observed a correlation between *DNMT3B* promoter polymorphisms, DNMT3B protein levels, and DNA methylation levels, suggesting that the increased risk of cancer observed in carriers of those polymorphisms might be due to impairments of DNA methylation in cancer cells [22,32]. 

Except the presence of thymoma, no other investigated MG clinical characteristic was associated with this polymorphism in our population. Interestingly, others evaluated the contribution of this polymorphism to the onset and progression of autoimmune thyroid disease, and found no association [33]. The observed lack of association of the study polymorphism with MG risk or age at onset does not exclude that other epigenetic biomarkers might be linked to the disease. Unfortunately, little is still known concerning epigenetic modifications in MG, as the few available studies are focused on DNA methylation in thymoma specimens [30]. Therefore, due to the absence of available genome-wide data, the role of DNA methylation in MG is still to be clarified. By contrast, several studies revealed global or gene specific hypomethylation, impaired activities of enzymes such as DNMTs or methyl CpG binding proteins, histone tail modifications, and/or changes in non coding RNAs, in autoimmune diseases such as systemic lupus erythematosus, rheumatoid arthritis, Sjögren’s syndrome, psoriasis, multiple sclerosis, and systemic sclerosis, among others [3,7,8], suggesting that further studies are needed to elucidate the possible contribution of epigenetics to the pathogenesis of MG. 

## Conclusions

The thymus plays distinct roles in the pathogenesis of the different AChR+ MG subtypes. Inflammatory, neoplastic and age-related alterations of the thymus are of pivotal relevance for the initiation of anti AChR autoimmunity in early onset MG, thymoma-associated MG and, likely, late onset MG, respectively [34]. At best of our knowledge the present is the first investigation of *DNMT3B* promoter polymorphisms in MG patients. Our study suggests that the presence of the *DNMT3B* -579T allele might represent a risk factor for the development of MG-associated thymomas, particularly in carriers of the homozygous TT genotype, while it probably does not have effect on other MG subtypes. Therefore, the analysis of this polymorphism could help to identify those AChR+ MG individuals at increased risk to develop a thymoma.
